# The Cost of Self‐Defense: Browsing Effects in the Rare Plant Species *Salix arizonica*

**DOI:** 10.1002/ece3.70582

**Published:** 2024-11-24

**Authors:** Shannon J. Lencioni, Rob Massatti, Ken Keefover‐Ring, Liza M. Holeski

**Affiliations:** ^1^ Department of Biological Sciences, Center for Adaptable Western Landscapes Northern Arizona University Flagstaff Arizona USA; ^2^ U.S. Geological Survey, Southwest Biological Science Center Flagstaff Arizona USA; ^3^ Department of Botany and Geography University of Wisconsin Madison Wisconsin USA

**Keywords:** browsing, optimal defense theory, phenolic glycosides, trade‐offs, willow

## Abstract

Coevolution between plants and their animal predators has led to diverse defensive adaptations. Multiple theories of defense propose that there are resource allocation costs associated with producing chemical defenses. One leading hypothesis, optimal defense theory (ODT), suggests that natural selection will result in the allocation of resources to defenses that optimize the cost‐to‐benefit ratio between defense and other functional processes. The population decline of the rare subalpine wetland species, Arizona willow (*Salix arizonica*), has been attributed to various biotic and abiotic factors, with browsing from wild and domestic ungulates as a significant concern for at least three decades. In a field experiment using natural populations, we compare the relationship between phytochemical defense and height in Arizona willows with and without long‐term protection from browsing via browse exclosures. Consistent with the predictions of ODT, individuals with physical protection from ungulate browsing for multiple years had significantly lower phenolic glycoside (PG) concentrations and increased plant height compared to unprotected individuals. A similar pattern was found across all individuals, whereby total PG concentration and height were negatively correlated. In a short‐term experiment in natural populations, changes in levels of defense were not observed when plants received protection for only one growing season. The contrasting pattern of defense plasticity in response to long‐term versus short‐term physical protection suggests a differential plastic response in this long‐lived species. Delayed reduction in PG concentration may serve as a benefit to avoid mismatches between environmental cues and responses. Our research sheds light on the intricate dynamics between plant‐defense strategies, environmental pressures, and evolutionary adaptations in shaping plant–browser interactions.

## Graphical abstract

Willows with physical protection from ungulate browsing for multiple years had significantly lower phenolic glycoside (PG) concentration and increased plant height compared to unprotected individuals. In contrast, changes in levels of defense were not observed when plants received protection for only one growing season. The contrasting pattern of defense plasticity in response to long‐term versus short‐term physical protection suggests a differential plastic response in this long‐lived species.

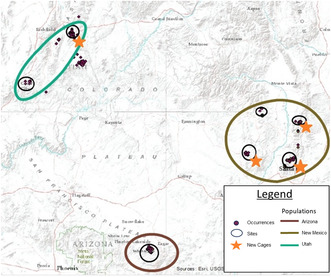

## 1 Introduction

Plants have coevolved through interactions with herbivores and have evolved a diversity of defense traits, both morphological (spines, thorns, and tough leaves) and chemical (secondary metabolites). Secondary metabolites are present in almost all plants (Wink 2003); they play diverse roles in plant defense (Aharoni and Galili 2011; Guerriero et al. 2018) and can lead to an increase in fitness and function (Aharoni and Galili 2011). While plant defenses can increase plant fitness by reducing herbivory (Huot et al. 2014), an inherent trade‐off arises assuming finite resources (McKey 1979; Rhoades and Cates 1976). Resources allocated to defense may reduce those allocated to other plant functions such as growth and reproduction (Huot et al. 2014). This resource allocation trade‐off may necessitate a prioritization between defense and growth, or defense and other life history traits (Coley 1983; He, Webster, and He 2022; Herms and Mattson 1992). Optimal defense theory (ODT) postulates a cost associated with the production of chemical defenses, suggesting that plants have evolved with different strategies for the allocation of resources to defenses that optimize the cost‐to‐benefit ratio (Fagerstrom, Larsson, and Tenow 1987).

Phytochemical defenses may be constitutive (plants will always have chemical defense compounds present) or induced (levels of chemical defense compounds increase in response to attack) (Karban and Myers 1989). In systems that experience high levels of herbivory, constitutive defenses should be favored for defense strategy (Bixenmann et al. 2016). However, assuming constitutive compounds are costly to make, the induction of secondary metabolites in response to attack may represent a cost‐saving strategy (Zangerl 2003). The presence and/or extent of induction is variable and is dependent on several factors, including plant identity (e.g., genotype or species), environmental inputs (nutrient availability and temperature), type of attacker, and timing of herbivory (Bowers and Stamp 1993; Eubanks, Carr, and Murphy 2005; Holton, Lindroth, and Nordheim 2003; Moreira et al. 2013; Moreira et al. 2015). Conversely, a strategy relying on inducible defenses can be more costly in the end, relative to constitutive defenses, resulting in more damage and reduction in photosynthetic material if induction of defense is delayed (Kirschbaum 2011; Xia et al. 2009). Numerous studies have found resource costs associated with increasing production of defense chemicals (Holeski et al. 2012; Koricheva 2002; Stolter et al. 2005), and this has been well documented across different plant lifeforms such as trees, forbs, and graminoids (Adler and Kittelson 2004; Kruger et al. 2020; Li et al. 2020).

**FIGURE 1:**
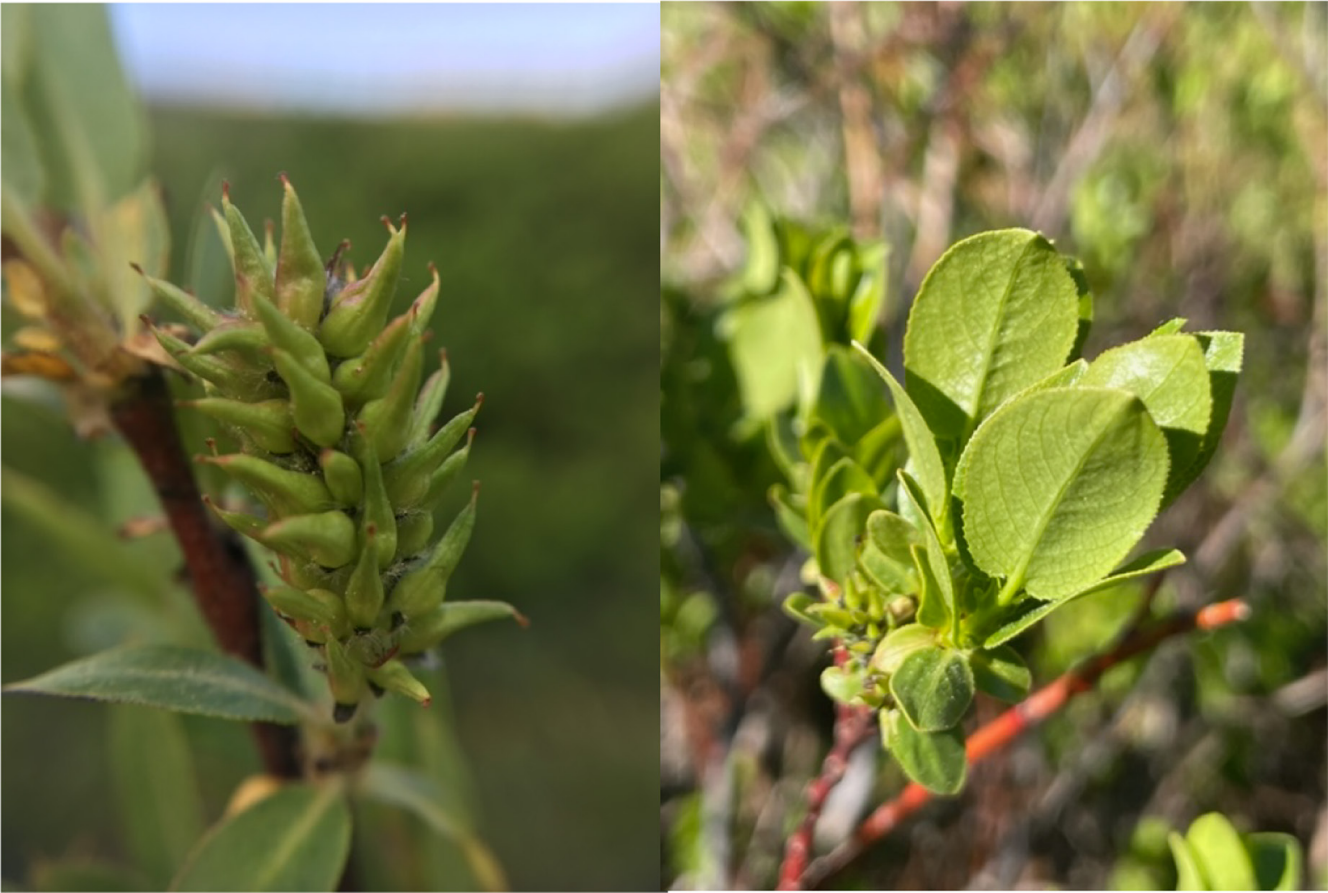
*Salix arizonica*. Female catkin on the left, leaves with distinct morphological characteristics on the right.

Arizona willow (*Salix arizonica*; Salicaceae; Figure 1) presents a compelling and novel opportunity to investigate the intricate relationship between defense and growth. This rare species is browsed heavily by ungulates throughout its range (Decker 2006; Maschinski 2000; Tonne 2002; Arizona Willow Interagency Technical Team 1995). Arizona willow is found in small populations that are geographically disjunct from each other, and it is a species of critical conservation concern because it is at high risk of extinction due to declining populations (NatureServe 2024; Arizona Willow Interagency Technical Team 1995). Ungulate browsing can reduce plant growth through the removal of leaves, as well as damage branches, change plant architecture, and limit reproduction by the removal of plant material (Maschinski 2000; Poveda et al. 2003). While Arizona willow may benefit from defense compounds that reduce browsing, ODT predicts a potential trade‐off between induced defenses and plant growth.

Arizona willow, like other members of the *Salix* genus, produces phenolic glycosides (PGs) (Julkunen‐Tiitto 1989; Tahvanainen et al. 1985; Keefover‐Ring et al. 2022). More than 20 different PGs have been found between members in the genus *Salix*, reaching concentrations of up to 30% of dry weight in plant tissue (Boeckler, Gershenzon, and Unsicker 2011). While secondary metabolites are variable across *Salix* species, the PGs salicortin and salicin emerge as widespread compounds (Boeckler, Gershenzon, and Unsicker 2011). The distribution of PGs can vary with hybridization, a frequent phenomenon within Salix (Orians 2000; Fogelqvist et al. 2015; Gramlich, Wagner, and Hörandl 2018; Hardig et al. 2000). The role of PGs as defense compounds has been well documented, particularly in the Salicaceae family, due to reducing the palatability of tissues toward herbivores (Bailey et al. 2007; Lindroth and Hwang 1996; Nichols‐Orians et al. 1992; Orians et al. 2003; Rehill et al. 2005; Rivas‐San Vicente and Plasencia 2011; Scriber, Lindroth, and Nitao 1991; Tahvanainen et al. 1985). Phenolic glycosides can reduce browsing susceptibility by ungulates, as seen in a study of intraspecific variation among *Populus tremuloides* (trembling aspen; Salicaceae), where clones with higher PG concentrations displayed reduced susceptibility to browsing relative to those with lower levels of PGs (Lastra, Kenkel, and Daayf 2017). Though the strategy of PG production differs across *Salix* species, PG levels are inducible in *Salix* in response to browsing (Fields and Orians 2006; Orians et al. 2010; Stolter et al. 2005). Despite the extensive history of plant defense research on willows (Danell et al. 1985; Hallgren et al. 2003; Orians et al. 2010), no prior studies have investigated the defense compounds of Arizona willow and how they interact with plant fitness and/or browse severity.

Our research assessed the relationship among phytochemical defense concentration, browsing levels, and growth across individuals and populations of Arizona willow. We hypothesize that, as ODT predicts, browsing will prompt plants to prioritize allocation of resources to defense compounds instead of growth. Our key objectives are to assess if: (1) long‐term protection from browsing reduces PG concentration in willows compared to those with long‐term exposure to browsers; (2) newly implemented, short‐term, protection from browsing allows plants to reduce investment in defense compounds; and (3) there is a trade‐off between willow growth and PG levels.

## 2 Methods

### 2.1 Species and Population Selection

Arizona willow is primarily found in subalpine wet meadows, stream sides, and cienegas (Long and Medina 2007) from 2530 to 3560 m elevation. The species occurs as a shrub that forms a prostrate mat, large hedge, or thicket, growing up to 2.6 m tall (https://swbiodiversity.org/seinet/). Arizona willow is a Forest Service Sensitive Species and holds a global rank of G2 (imperiled), facing the imminent risk of extinction or collapse due to its restricted range and limited population occurrences (USDA Forest Service 2016; NatureServe Conservation Status 2024).

Despite variable and sparse population monitoring by land managers, it is evident that Arizona willow is in decline (Tonne 2002). Browsing, disruption of hydrologic processes, disease, timber harvest, and recreation are all listed as leading threats to the decline of willow populations within the Arizona Willow (*Salix arizonica*) Conservation Agreement and Strategy (Arizona Willow Interagency Technical Team 1995).

In the state of Arizona, Arizona willow is protected under the Arizona Native Plant Law as a Highly Safeguarded Species (Arizona Revised Statutes 1999). Since the mid‐1990s, land managers in the Arizona White Mountains have made significant efforts to protect it by installing a variety of ungulate exclosures around most occurrences. Though the species was first described with specimens from the White Mountains (Dorn 1975), by the time of this study the species distribution was well documented in Utah, northern New Mexico, and southern Colorado (Arizona Willow (*Salix arizonica*) Conservation Agreement and Strategy; Decker 2006; Tonne 2002). Previous molecular research comparing populations from Utah and Arizona found that there is genetic dissimilarity between the two populations, but the lack of a significant correlation of genetic similarity and geographic distance suggests there is recent gene flow (Thompson 2003). Preliminary molecular analysis (Massatti unpublished results) supports that Utah populations are genetically dissimilar to Arizona populations and that there is evidence of hybridization within the Utah populations, particularly with Booth's willow (*S. boothii*). Additionally, populations that occur in northern New Mexico and southern Colorado are genetically dissimilar from Utah and Arizona populations, with less hybridization occurring. There is some evidence that these populations share an evolutionary history extending back to the Neogene period (23–2.6 million years ago), when the young volcanic highlands where Arizona willow occurs were connected with the ancient Colorado River tributaries (Long and Medina 2007). Currently, populations are isolated in high‐elevation pockets surrounded by vast arid landscapes ranging from 160 to 515 km from each other.

For this study, we collected samples from individuals that contained morphological characteristics belonging to Arizona willow as described by Dorn (1975) and where preliminary molecular research agrees that populations are in fact Arizona willow. The leaves are the most notable morphological feature, glabrous on both sides but can be pilose when young, while mature blades are elliptic to broadly elliptic, the margins glandular dotted to serrulate, and the base cordate to rounded (Dorn 1977). Dioecious and coetaneous, the capsules on the female catkins are glabrous, subtended by brown–black hairy bracts (Welsh et al. 2016). Dorn (1975) conducted a systematic study of the subsection *Cordatae* and found that flavonoid compounds provided the most useful taxonomic characteristic of willows, where Arizona willow was distinctly different from the closely related Booth's willow.

### 2.2 Field Sampling

**FIGURE 2:**
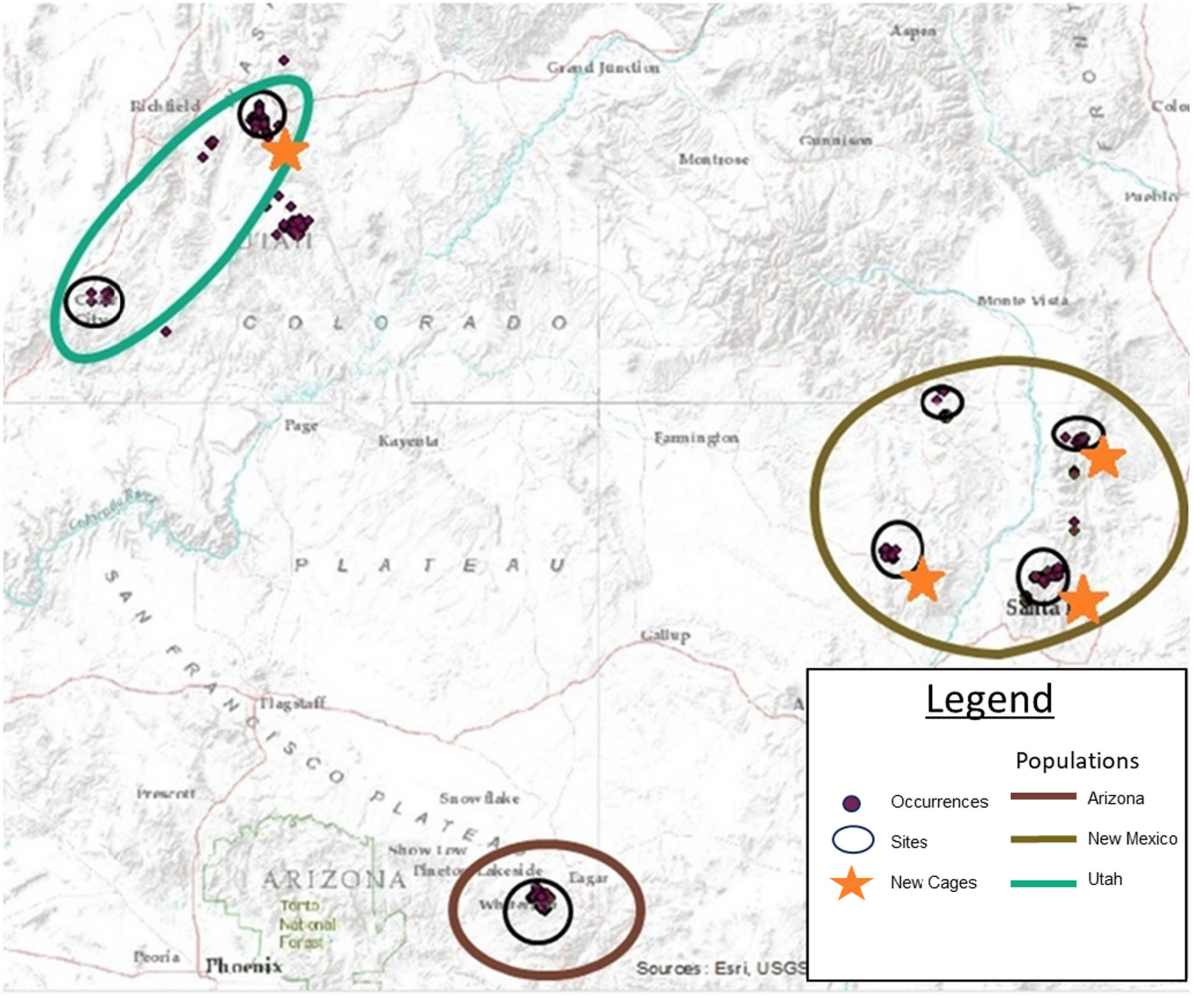
Map of Arizona willow distribution. Populations are outlined in larger colored polygons. Collections sites and new caging locations provided.

During the growing season of 2022 (late May through September), we collected foliar tissue from Arizona willow samples throughout its range. For sampling, the distribution of Arizona willow was broken into three distinct populations by genetic similarity (Thompson 2003) and geographic distances, named as Arizona, New Mexico (containing all collections within Colorado and New Mexico), and Utah (Figure 2). Within populations we determined collection sites (Figure 2), and within collection sites we sampled leaves from subsites (Table A1). When possible, we collected tissue from 10 to 20 individuals per subsite with a 5–15 m distance between individuals to reduce the possibility of recollecting from the same individual. Identification of the exact area of one individual can be difficult to delineate in this clonal species; thus, collecting across this distance gap eliminated the chances of inadvertently recollecting from the same individual. We haphazardly collected three to six leaves per shrub for assessment of secondary chemical constituents. These leaves were collected from both current and past‐season stems to eliminate morphological age as an effect. Individuals were marked with flagging to allow for resampling.

We took additional measurements from each individual, including plant height (the length of the longest primary stem from tip to the ground) and basal stem diameter of the largest shoot of an individual. Sex of the individual was noted when possible since secondary compounds can vary between sexes (Palo 1984). The phenology of the individual was documented as well as notable leaf characteristics such as evidence of disease or yellowing, substantial insect damage, or the presence of rust. We noted browsing severity categorically as: none, mild, moderate, or heavily browsed. We determined browsing severity with a visual assessment of the proportion of browsed stems versus unbrowsed stems of an individual, where 25% or less of browsed stems was categorized as mild, 26%–50% was moderate, and anything more than 50% browsed was heavy. Foliar samples were stored and dried in coin envelopes in silica.

To assess the effect of long‐term protection on PG concentrations in Arizona willow (Objective 1), we focused our collections within the Arizona population; this included individuals in preexisting browse exclosures. These exclosures were initially installed by the Forest Service in 1995 throughout several subsites and have been maintained for almost three decades to accommodate plant growth. Foliar tissue samples were haphazardly collected from 50 individuals throughout nine subsites, with samples from 29 caged individuals and 21 uncaged individuals. Most caged individuals had low levels of browse when stems were reachable for ungulates; only eight caged individuals had no evidence of browse.

To assess whether new, short‐term protection would result in plants reducing concentrations of defense compounds (Objective 2), we constructed new temporary exclosures at the end of the 2022 growing season at locations that did not contain functional exclosures (Figure 1). Locations were chosen where individuals were heavily browsed in 2022. We built new exclosures at several subsites within three sites in northern New Mexico and one site in Utah. Across the four sites, a total of 35 individuals were protected with new exclosures.

Resampling took place at the end of the 2023 growing season (August) to allow for the likelihood of browsing to occur on unprotected individuals. Due to weather or browsing, flagging was lost for most of the unprotected individuals, so we sampled new individuals that had evidence of current season browsing (ungulate damage on young green stems) in place of previous year samples to compare with current season protected plants. Within the New Mexico population, some exclosures were impacted by ungulates and/or weather and were essentially destroyed at two different collection subsites: Laguna Saddle South and Rio de las Vacas. Some protected individuals were consequentially browsed, so we sampled new, comparable individuals (that didn't have current season browsing evidence) nearby.

### 2.3 Quantification of Phenolic Glycosides

For phytochemical quantification, we extracted and analyzed compounds with high‐performance liquid chromatography (HPLC; Agilent 1260 HPLC with a diode array detector and Poroshell 120 EC‐C18 analytical column [4.6 × 250 mm, 2.7 μm particle size]; Agilent Technologies, Santa Clara, California, USA) maintained at 30°C. Dry tissue samples were ground to a fine powder using one 5 mm stainless steel bead with a dental amalgamator for approximately 20 s. We weighed out samples (between 5.0 and 7.0 mg) and added 500 μL of methanol to each sample before placing them in a sonicator for 20 min. Samples were left overnight (15–16 h) at room temperature to allow for full extraction of compounds. Samples were centrifuged, and 100 μL of the aliquot was pipetted into an HPLC glass insert. We then placed them in a speedvac for 12 min on low to evaporate methanol from the aliquot. Once inserts were dry, we added 100 μL of internal standard (1.0 mg of 98% Phenyl beta‐D‐glucopyranoside in 1.0 mL of DI water) and vortexed samples for 10 s each. HPLC run conditions included a binary mobile phase gradient with DI water at pH 2.6 with phosphoric acid as mobile phase A and acetonitrile as mobile phase B at a constant total flow rate of 1.0 mL/min. The 30‐min gradient for each run consisted of B initially set at 5% and increasing 5% every 3 min until it reached 95%. We injected 3 mL of the standards and samples, monitored ultraviolet signals at 200 (internal standard) and 274 (phenolic glycosides), and used a diode array detector to collect ultraviolet data from 190 to 360 nm. We calculated chemical constituent concentrations as salicin equivalents and as milligrams per foliar dry weight (grams).

Arizona willow samples contained up to five phenolic glycosides, where salicin (C_13_H_18_0_7_), salicortin (C_20_H_24_O_10_), and HCH‐salicortin (C_27_H_29_O_13_) were consistently the most abundant compounds (Kruger et al. 2020). Additionally, two uncharacterized compounds were frequently detected, although their values often fell below the detection limit. In cases where the HPLC was unable to capture values for these unknown compounds (including a few instances for HCH‐salicortin), we recorded a zero instead of leaving the entry blank. We used this approach to avoid reducing the sample size for subsequent analyses involving these specific compounds.

### 2.4 Statistical Analysis

We conducted statistical analyses using R‐Studio version 4.3.0 (RStudio Team 2023). To assess the relationship(s) between phenolic glycosides (PGs) and predictors such as browsing and caging effects, we utilized a combination of linear regression and linear mixed‐effects models. We fitted the linear mixed‐effects models using the ‘lme4’ package (Bates et al. 2015). To visualize results, graphics were generated using the ‘ggplot2’ package (Wickham 2016).

#### 2.4.1 Objective 1: Effects of Long‐Term Protection on Defense

A mixed‐effects linear model was used to explore if long‐term protection had a significant effect in reducing PG concentrations in individuals relative to those that were not protected. After checking assumptions, we created separate linear mixed‐effect models for each PG concentration as a response variable separately, as well as a model using the total sum of PG compounds together. For these analyses, samples from only the Arizona site were included because this was the only site that had undamaged structures around a subset of individuals over a long period of time (20 years or more). For model selection, we also included an interaction term of browse severity × cage, but because this term was not significant across all compounds modeled, we excluded it from subsequent models.

$$\mathrm{PG}=\text{browse severity}+\text{cage}+\left(1|\text{subsite}\right)$$

#### 2.4.2 Objective 2: Effects of New (Short‐Term) Protection on Defense

Arizona willow individuals were clustered into ‘populations’ that were most genetically similar with one another (Arizona, Utah, and New Mexico) and used in the creation of simple linear models to explore the question of whether short‐term protection from browsing had a significant effect on PG concentrations. To avoid overfitting our models due to the small sample size, we proceeded using simple linear models over mixed‐effects. As in the analysis for long‐term browsing effects, we created separate models for each compound as a response variable and with the total sum of PG compounds together, with cage and population as fixed effects. For model selection, we included an interaction term of population × cage, but because this term was not significant across all modeled compounds, we excluded it from subsequent models.

PG=cage+population

#### 2.4.3 Objective 3: Trade‐Offs between Growth and Defense

We used populations as described above and created linear mixed‐effects models using the predictors ‘site’ and ‘subsite’ as random effects. Height was used as the response variable to test if PG production has a significant effect on plant growth. To meet assumptions, we log transformed response variable. For model selection, we initially included the following interactions terms: browse severity × PGs, browse severity × population, browse severity × cage, PGs × population, population × cage, and PGs × cage. AIC determined the model without interaction terms to be the best fit (Table A2), thus we continued by using the models without an interaction effect.

$$\begin{array}{c} \log\left(\text{height}\right)=\text{browse severity}+\mathrm{PGs}+\text{population}\\ \;+\text{cage}+\left(1|\text{site}/\text{subsite}\right) \end{array}$$

## 3 Results

### 3.1 Effects of Long‐Term Protection on Defense

**FIGURE 3:**
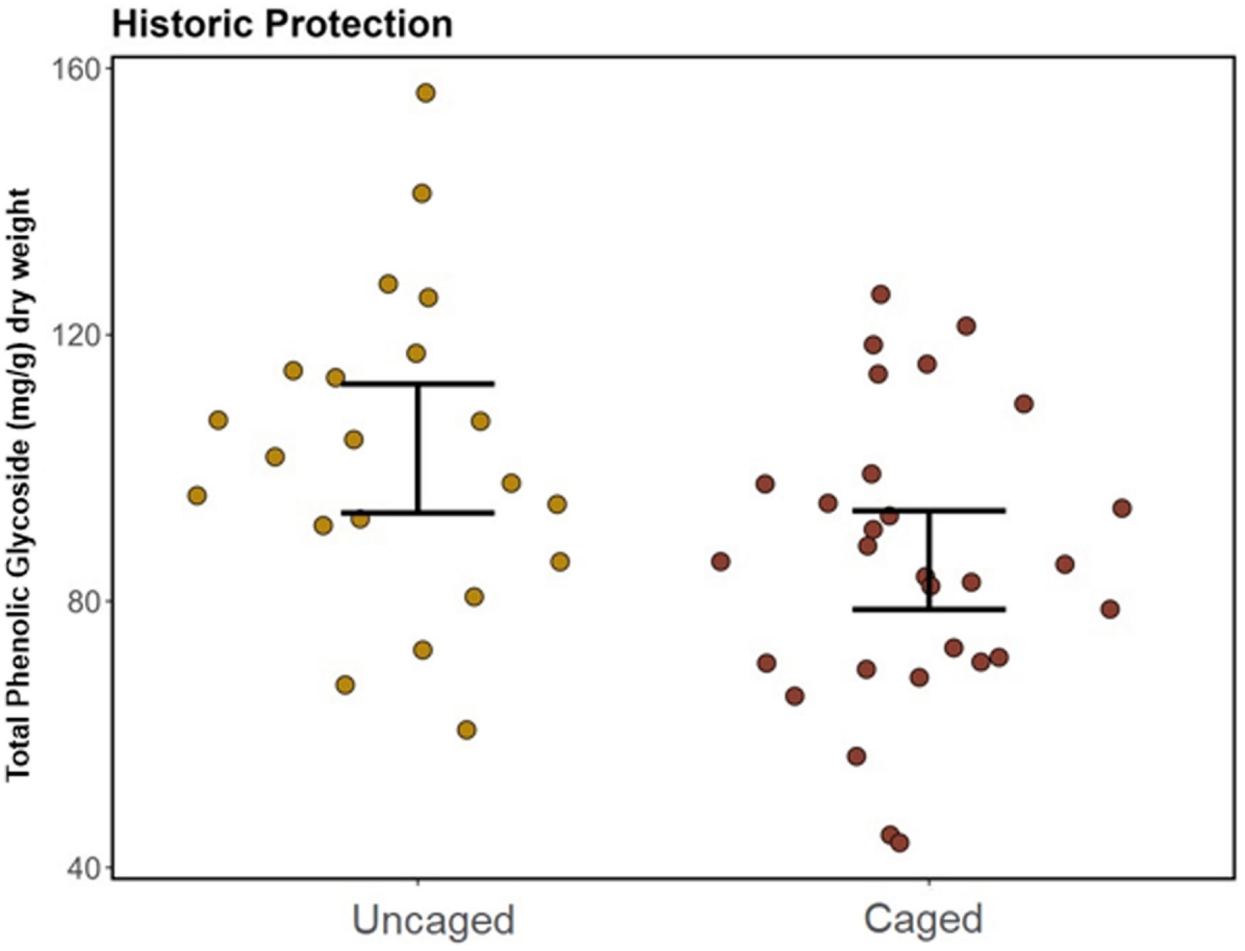
Total PG concentration mg/g plant dry weight by protection category for the Arizona population. Each dot is the concentration of one individual. 95% confidence intervals of the mean for each category displayed with black bars.

Phenolic glycoside levels were significantly reduced in plants with long‐term protection relative to those without long‐term protection in the Arizona population (Figure 3, Table 1). Individuals that had long‐term protection had total PG concentrations that were on average 25.8 mg/g dry weight lower than unprotected individuals (Figure 3). The concentrations of most individual PGs were also reduced in these plants (Table 1). For both comparisons, the effects of long‐term protection were independent of recent (current season) browsing activity. Current season browsing activity did not have a significant effect on individual or total PG concentrations (Table 1). No significant interaction effects were found in the models, indicating that populations have a similar response in PG concentration regardless of browse severity and cage effect.

**TABLE 1:** Results of mixed‐effects linear models showing difference in total and individual PG concentrations between long‐term protected (caged) versus unprotected (uncaged) individuals from the Arizona population.

Model	Factor	Estimate	*T*‐value	*p*
Total PGs = cage + browse severity	**Cage**	**−25.817**	**−2.370**	**0.024**
	Browse severity—Mild	7.149	0.713	0.450
	Medium	−6.622	−0.530	0.599
	Heavy	−8.614	−0.648	0.520
Salicin = cage + browse severity	Cage	−0.614	−0.921	0.363
	Browse severity—Mild	−0.354	−0.649	0.519
	Medium	−0.574	−0.826	0.413
	Heavy	0.023	0.032	0.975
Salicortin = cage + browse severity	**Cage**	**−23.395**	**−2.336**	**0.026**
	Browse severity—Mild	8.325	0.889	0.379
	Medium	−4.571	−0.392	0.697
	Heavy	−8.280	−0.667	0.509
HCH‐Salicortin = cage + browse severity	**Cage**	**−0.840**	**−2.200**	**0.033**
	Browse severity—Mild	−0.187	−0.509	0.613
	Medium	−0.395	−0.854	0.398
	Heavy	−0.252	−0.508	0.614
Unknown 1 = cage + browse severity	Cage	−0.582	−1.350	0.188
	Browse severity—Mild	−0.389	−1.014	0.316
	Medium	−0.696	−1.475	0.147
	Heavy	−0.353	−0.709	0.482
Unknown 2 = cage + browse severity	Cage	−0.069	−0.578	0.567
	Browse severity—Mild	−0.007	−0.066	0.948
	Medium	−0.060	−0.436	0.665
	Heavy	−0.133	0.912	0.367

*Note:* All models included subsite as a random effect. Compounds that showed significant differences between treatments are in bold text. Sample size = 50.

### 3.2 Effects of New (Short‐Term) Protection on Defense

**FIGURE 4:**
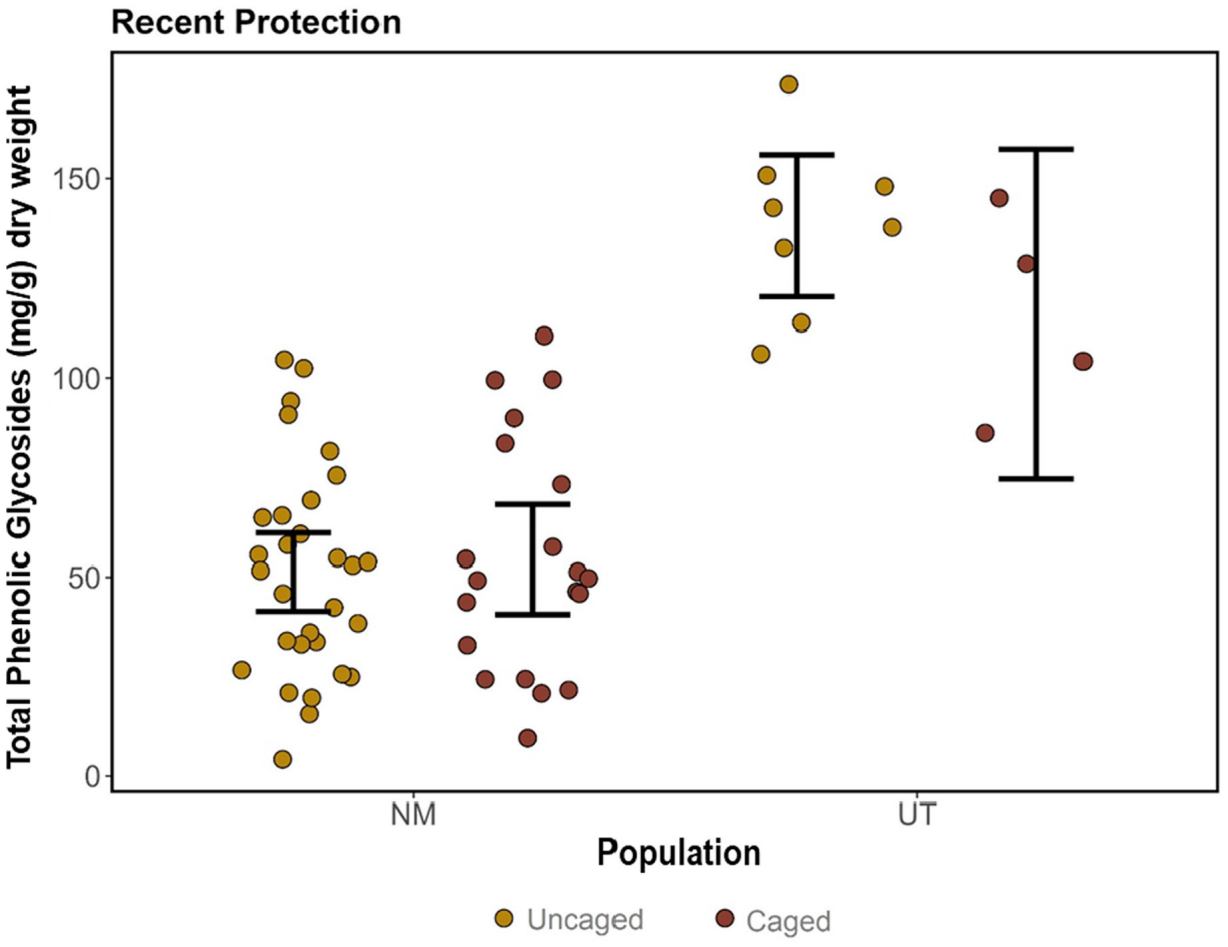
Total PG concentration mg/g plant dry weight by protection category per population. Each dot is the concentration of one individual. 95% confidence intervals of the mean for each category displayed with black bars.

**FIGURE A1:**
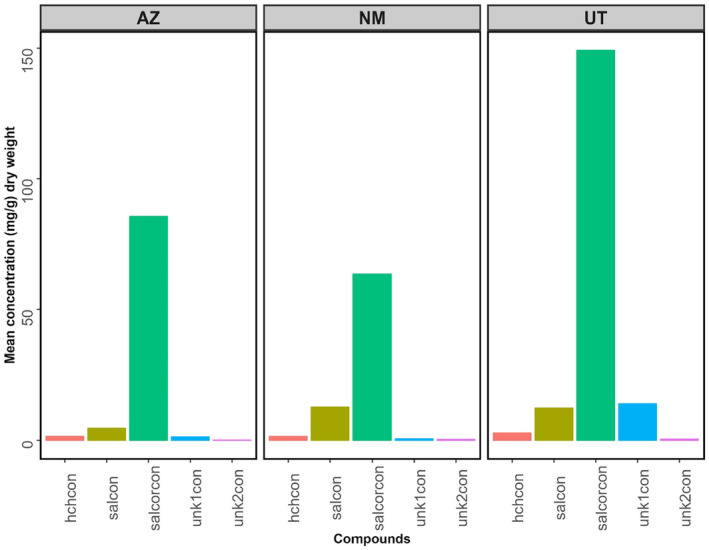
Mean concentration of phenolic glycoside compounds (mg/g) dry weight by populations where compounds are labeled as: hchcon, HCH‐Salicortin; salcon, salicin; salcorcon, salicortin; unk1con, unknown PG 1; unk2con, unknown PG 2.

We did not observe a significant reduction in PG concentration when newly protected plants, which did not have long‐term caging and thus experienced historical browsing events, were provided protection from browsing events within one growing season (Figure 4 and Table 2). This pattern was similar across both the New Mexico and Utah populations where we installed the short‐term protection. While defense concentrations differed substantially across populations (Figure A1), populations responded similarly to the short‐term protection (Figure 4 and Table 2). There was not a significant interaction effect between new protection and population, indicating that populations responded similarly to the new protection.

**TABLE 2:** Results of linear models showing difference in total and individual PG concentration between short‐term protected (caged) versus unprotected (uncaged) individuals from the New Mexico and Utah populations.

Model	Factor	Estimate	*T*‐value	*p*
Total PGs = cage + population	Caged	−4.295	−0.617	0.540
	Population—UT	77.739	8.940	< 0.001
Salicin = cage + population	Caged	−1.015	−1.631	0.108
	Population—UT	7.058	9.079	< 0.001
Salicortin = cage + population	Caged	−2.416	−0.385	0.701
	Population—UT	62.214	7.948	< 0.001
HCH‐salicortin = cage + population	Caged	−0.063	−0.260	0.796
	Population—UT	2.460	8.067	< 0.001
Unknown 1 = cage + population	Caged	−0.791	−1.296	0.200
	Population—UT	5.852	7.679	< 0.001
Unknown 2 = cage + population	Caged	−0.009	−0.133	0.895
	Population—UT	0.155	1.818	0.074

*Note:* Total and individual compounds did not show a significant difference between the two populations. Sample size = 62.

### 3.3 Trade‐Offs between Growth and Defense

**FIGURE 5:**
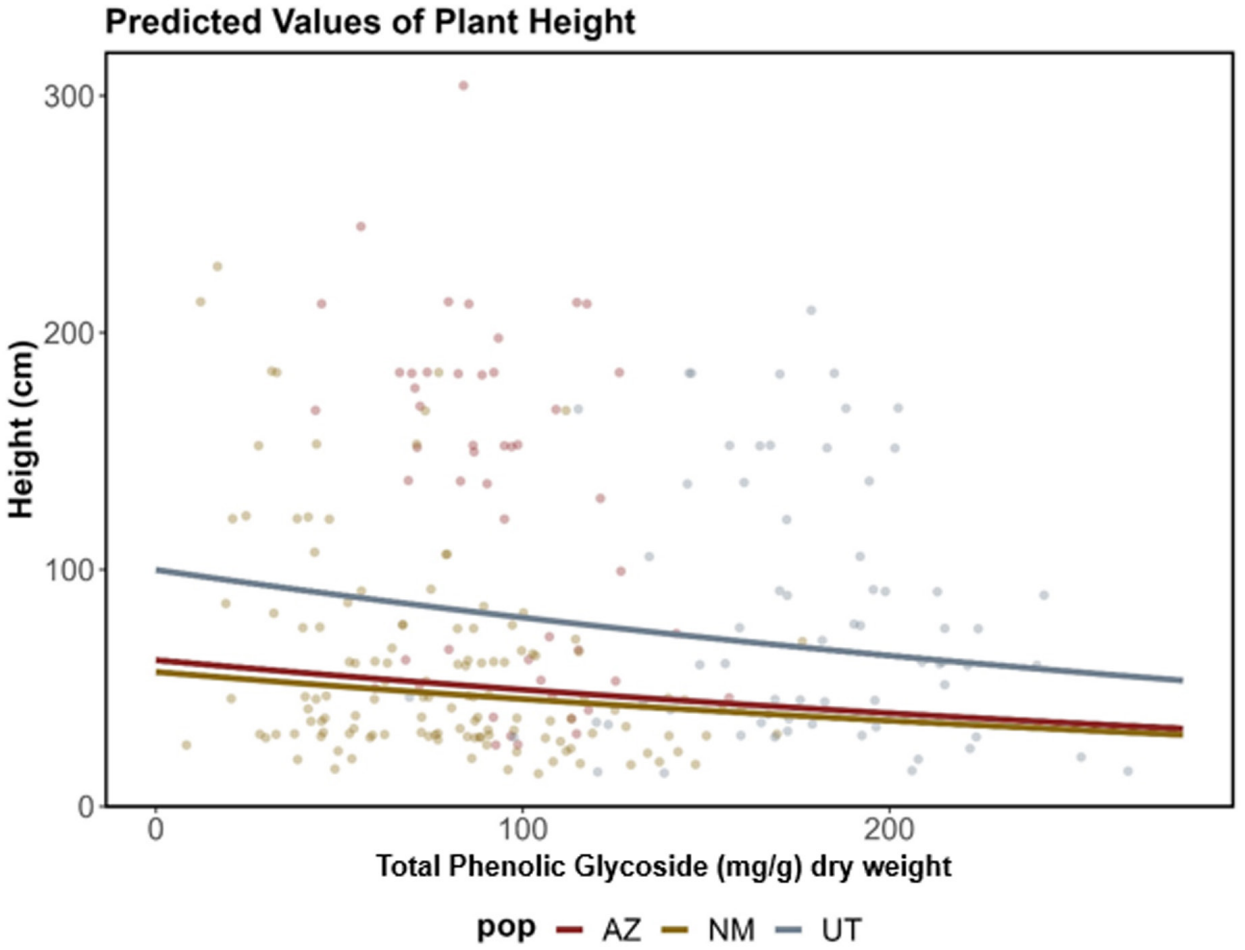
Model predictions of plant height based on total PG concentrations, as measured in mg/g per foliar dry weight.

Across all populations, plant height was significantly and positively related to the presence/absence of long‐term protection (ungulate exclosures). Plant height was negatively related to the concentration of total PGs as well as the concentration of salicortin (Table 3). Caged individuals were an average of 119.7 cm taller in plant height compared to unprotected individuals. With each 10 mg/g foliar dry weight increase in total PG concentration, there was a 2.43‐cm decrease in plant height (Figure 5 and Table 3). These effects of long‐term protection were consistent regardless of recent (current season) browsing activity, which did not have a significant effect on individual plant height (Table 3). Plant population did not have a significant effect on height (Table 3), despite these populations being genetically distinct from one another.

**TABLE 3:** Results of mixed‐effects linear models showing predictions of total and individual PG concentrations on plant log(height), representing sampling that occurred throughout the species distribution.

Model	Factor	Estimate	*T*‐value	*p*
log(height) = total PGs + cage + population + browse severity	**Total PG**	**−0.002**	**−2.350**	**0.020**
	**Caged**	**1.110**	**5.645**	**< 0.001**
	Population—NM	−0.084	−0.204	0.848
	UT	0.481	1.055	0.348
	Browse severity—Mild	0.155	1.106	0.270
	Moderate	0.170	1.168	0.244
	Heavy	−0.072	−0.472	0.637
log(height) = salicin + cage + population + browse severity	Salicin	−0.002	−0.534	0.594
	**Caged**	**1.133**	**5.741**	**< 0.001**
	Population—NM	−0.033	−0.075	0.944
	UT	0.303	0.638	0.558
	Browse severity—Mild	0.145	1.021	0.308
	Moderate	0.192	1.314	0.190
	Heavy	−0.064	−0.414	0.679
log(height) = salicortin + cage + population + browse severity	**Salicortin**	**−0.002**	**−2.167**	**0.031**
	**Caged**	**1.104**	**5.599**	**< 0.001**
	Population—NM	−0.107	−0.258	0.809
	UT	0.435	0.955	0.392
	Browse severity—Mild	0.160	1.138	0.256
	Moderate	0.176	1.210	0.228
	Heavy	−0.069	−0.453	0.621
log(height) = HCH‐salicortin + cage + population + browse severity	HCH‐salicortin	−0.0464	−1.710	0.089
	**Caged**	**1.118**	**5.634**	**< 0.001**
	Population—NM	−0.059	−0.142	0.894
	UT	0.358	0.795	0.471
	Browse severity –Mild	0.160	1.133	0.258
	Moderate	0.180	1.237	0.217
	Heavy	−0.073	−0.474	0.636
log(height) = unknown 1 + cage + population + browse severity	Unknown 1	−0.0160	−1.803	0.073
	**Caged**	**1.187**	**5.923**	**< 0.001**
	Population—NM	−0.026	−0.061	0.954
	UT	0.512	1.061	0.342
	Browse severity—Mild	0.154	1.094	0.275
	Moderate	0.198	1.367	0.173
	Heavy	−0.054	−0.356	0.722
log(height) = unknown 2 + cage + population + browse severity	Unknown 2	−0.067	−1.189	0.236
	**Caged**	**1.123**	**5.668**	**< 0.001**
	Population—NM	−0.067	−0.072	0.946
	UT	−0.031	0.671	0.539
	Browse severity—Mild	0.149	1.051	0.294
	Moderate	0.196	1.343	0.181
	Heavy	−0.076	−0.495	0.621

*Note:* Predictions of long‐term protection treatment (caging) is also shown for each compound modeled. All models include site and subsite as random effects. Compounds that showed significant effects on plant height are in bold text. Sample size = 246.

## 4 Discussion

Most research on defense compounds has either been conducted through greenhouse or common garden experiments or, if field‐based, covers only a sample of the total geographic distribution of a species instead of encompassing its whole range. In contrast, this research was conducted with only field collections and covered most of the geographic extent of Arizona willow. We explored how browsing pressure influences defense by assessing the effects of both long‐term and short‐term protection on PG concentrations. Arizona willow had significantly lower PG compound concentrations, on average, when they had been provided adequate physical protection for multiple decades relative to unprotected plants. Additionally, long‐term protection had a positive effect on plant height that was not directly influenced by browse level. We found that there was no difference in PG concentrations for individuals that were newly protected from browsing in the short term (for one year). Finally, an increase in PG levels is correlated with decreased plant height, suggesting a trade‐off between growth and defense levels within the species. These findings provide insights on the broader ecological impacts of browsers on this species with respect to allocation to growth versus defense.

### 4.1 Effects of Long‐Term Protection on Defense

While browsing occurred on most long‐term protected individuals in the White Mountains of Arizona, there was a significant reduction in PG concentrations for protected individuals relative to unprotected individuals. Individuals that were provided long‐term protection experienced a 16.6% decrease in total PG compound concentration on average relative to unprotected individuals. This implies that this species induces defense chemicals in response to herbivory, as PG concentrations were significantly higher across uncaged and more readily browsed individuals. This phenomenon aligns with previous studies on other willow species, which have demonstrated an induction in PG compound concentrations in response to increasing browsing pressures (Fields and Orians 2006; Orians et al. 2010; Stolter et al. 2005).

Previous studies have also noted the effects of defense compounds on herbivore selection (Heiska et al. 2007; Holeski et al. 2012; Mason et al. 2010; Moore and Foley 2005), where there were higher rates of herbivory on plants that had reduced defense chemical concentrations. In quaking aspen (*Populus tremuloides*), another member of Salicaceae, when elk were introduced to a long‐term protected clonal population, there was an elimination of 60% of the population (Bailey et al. 2007). Saplings with a 2% concentration of the PG tremulacin experienced an 80% chance of mortality, but with only a 6% increase in tremulacin concentration, the chance of mortality was reduced to 5% (Bailey et al. 2007).

We found a significant effect of long‐term protection on defense levels despite this being a study in natural populations of willow where we were not able to standardize across several sources of potential variation known to influence levels of defense compounds, including sex and age. We were not able to statistically assess the relationship between PG production and plant sex in this study, as we were unable to access every plant during its reproductive period (or plants did not have reproductive tissue) for over half of the samples used for our model predictions. Observationally, there was a higher occurrence of reproductive tissue in plants in long‐term exclosures; we were able to determine the sex of 86% of these individuals. Sexual dimorphism is known to affect levels of phytochemical defense as well as growth rate and other life history traits in other willow species, with females frequently having higher levels of foliar defenses (Palo 1984; Boecklen et al. 1990) though some species express differences in some compounds between sexes though they may contain similar total concentrations, making one sex more susceptible to browsing (Moritz et al. 2018). Other studies have shown that in several species of willows, males have higher PG concentrations than their female counterparts (Keefover‐Ring et al. 2022), where this defense prioritization decreases their growth rate (Yang et al. 2020). A meta‐analysis also suggests that the previous assumption of females containing more defense compounds than males may not be as prominent as previously suggested (Sargent and McKeough 2022).

Plant age can also have substantial effects on defense compound production. Younger plants typically contain their highest concentration of secondary metabolites when they are more susceptible to fatality (Coley 1983; Cronin and Hay 1996; Van Alstyne et al. 1999). Within Salicaceae, quaking aspen growth between one and five years resulted in decreased PG concentrations that continued to decrease with age (Donaldson, Kruger, and Lindroth 2006). Even within seedlings that ranged in age only from five to nine weeks, PG concentrations were higher in younger seedlings (Orians et al. 2010). In our study, plant age was not included in models because of the difficulties in determining plant age for a shrub. However, incorporating the differences of ontogeny and dioecy (Danell et al. 1985; Elmqvist et al. 1991) would likely increase the strength of the signal of long‐term protection and potentially offer additional insight into the browsing susceptibility of Arizona willow populations.

### 4.2 Effects of New (Short‐Term) Protection on Defenses

In contrast to the significant reduction in PG concentrations observed with long‐term protection, short‐term protection (protection provided for one year) had no significant effect on total or individual PG concentrations. Browsing on unprotected individuals was evident across all field sites where new protection was constructed. We did find year‐to‐year variation in defense; for individuals sampled both years, there was a 5%–60% decrease in PG concentration from 2022 to 2023 (Figure A1). This plastic response was not attributed to new protection from browsing and thus is likely due to unmeasured environmental factors. Though there is a plethora of evidence that herbivory increases defense chemistry in a wide array of plant species (Adler and Kittelson 2004; Koricheva 2002; Stolter et al. 2005; Tahvanainen et al. 1985), little research has examined the reversibility of defense responses of individuals once herbivory is removed, which has been a critique in an overview of plasticity on secondary metabolites (Metlen, Aschehoug, and Callaway 2009).

### 4.3 Plant Response to Long‐ versus Short‐Term Exclosures

The lack of reduction in PG concentrations in willows under short‐term protection provides an intriguing contrast to the significant plastic reduction in PGs in willows under long‐term protection. Plasticity of defense compounds (including phenolic glycosides) in response to abiotic and biotic factors has been well documented in quaking aspen (Cope et al. 2021; Lindroth et al. 2007). Possible mechanisms for the observed lack of reduction in defense in willows under short‐term protection are genetic canalization or long lag time intervals between the environmental signal and the plant response. With genetic canalization, a genome consistently produces the same phenotype despite environmental variations or genetic perturbations (Hallgrimsson et al. 2019). In either mechanistic scenario, it may take longer than one season for plastic responses to be expressed in response to increased or reduced browsing pressures. This time delay was observed with Mountain birch (*Betula pubescens*), where the reversal of a defense induction took place within a two‐year timeframe (Kaitaniemi, Neuvonen, and Nyyssönen 1999).

Long‐lived perennial plants, such as shrubs and trees, have traditionally been associated with traits of irreversibly plastic genotypes (Utz et al. 2014). Examples of irreversible traits can be seen in long‐lived systems with shade adaptation, where select branches on trees develop larger and more numerous buds in sunnier patches (Sprugel, Hinckley, and Schaap 1991). However, in other plant systems, such as herbaceous plants, rapid relaxation of plastic responses can occur within days or even hours following an herbivore attack, depending on the secondary metabolite mechanism (Turlings et al. 1995; Turlings and Tumlinson 1992; Underwood 1998). It is conceivable that for long‐lived species, the process of reverting a defense response may entail higher costs in plant fitness than we currently understand. On the other hand, a time lag or threshold for environmental change before plastic response can be advantageous for a species inhabiting a constantly changing environment. A delay or threshold could help prevent potential mismatches between an induced plastic response and an environment that reverts to a different state (Padilla and Adolph 1996). This observation is consistent with ODT, whereby rapid reversibility associated with short‐lived plants might be more costly for long‐lived species.

We were not able to assess the effects of long‐term and short‐term browse protection across all populations or to compare these effects within populations. We hypothesize that patterns of induction will be generally consistent across populations, but with possibly significant variation in response. Other studies of phytochemical defense plasticity across populations have shown variation in extent of response, but rarely are there patterns of presence vs. absence of induction (Agrawal, Gorski, and Tallamy 1999; Holeski et al. 2013).

### 4.4 Trade‐Offs Between Growth and Defense

We found significant trade‐offs between plant growth and defense production in Arizona willow that were consistent across populations. Model predictions demonstrated that increasing PG concentrations correspond to a reduction in plant height. This is consistent with ODT, which proposes a resource allocation trade‐off between defense and other life history aspects. The compounding effects of browsing and defense resource cost on plant height can have a biologically significant impact on plant fitness by reducing overall growth and reproductive capacity. Within Arizona willow, it appears that this trade‐off is partially driven by the PG salicortin, which consistently appeared in the highest concentration (Figure A1), though each PG had a negative relationship with plant height. Studies in closely related species to Arizona willow have similarly illustrated the trade‐off between plant growth and defense levels. External application of phenolic precursors illustrated a growth trade‐off in Laurel leaf willow (*S. pentandra*) by increasing PG concentration and decreasing plant growth without the use of mechanical tissue removal (Ruuhola and Julkunen‐Tiitto 2003). An increase of condensed tannins by 10% dry weight was associated with a nearly 40% decrease in quaking aspen in a controlled environment (Cole et al. 2016).

Growth, or plant height in the context of this study, can be considered a proxy for reproductive fitness in this long‐lived species. Eighty‐six percent of the long‐term protected individuals (generally tallest in this study) were reproductive, compared to 24% of unprotected individuals. The removal of reproductive tissues resulted in a reduction of future reproductive potential within Utah willow (*S. rigida*) but resulted in a rapid increase in overall biomass (Elmqvist et al. 1991). Studies in other systems, such as Cerrado trees (*Stryphnodendron adstringens*), have shown that plants that invest more resources into defense compounds may invest less in reproduction (Tuller et al. 2018). Likewise, a study comparing two different genotypes of lima bean (*Phaseolus lunatus*) found that the genotype with higher defense compounds had lower biomass and seed production (Ballhorn et al. 2014).

Of particular concern with browsing in this species is the potential impact on the ability of individuals to reach sexual reproduction. Many Arizona willow individuals that were not adequately protected from browsing were never observed with reproductive organs over the course of four growing seasons from years 2020 to 2023 (Lencioni personal observations). This pattern has also been noted in government species status reports (Tonne 2002). This is of major concern since Arizona willow does not appear to produce subterranean rhizomes (Maschinski 2000; Tonne 2002), which is an important dispersal strategy for other willow species (Pan and Price 2002). However, Arizona willow is thought to be a clonal species from above‐ground propagation, where it's known that propagation from stem cuttings is successful in the greenhouse setting (Maschinski 2000). The compounding cost of resources being allocated to defense along with the loss of above‐ground biomass may be resulting in a reduction of reproductive output and dispersal by Arizona willow, contributing to population declines.

### 4.5 Population Differences on Plant Defenses

The Utah population had significantly higher concentrations of PGs relative to the other two populations regardless of level of recent browsing (Figure A1). This could be due to genetic differences between populations, as the Arizona willow in Utah are often hybrids between Arizona willow and Booth's willow (Thompson 2003; Massatti unpublished results). Hybridization can have a multitude of different effects on defense compounds depending on the genetic architecture of the defenses. Defense concentrations can decrease (Court, Pocs, and Hendel 1992; Fahselt and Ownbey 1968), increase relative to parent concentrations (Spring and Schilling 1989), or can be similar to the parents' chemistry (Fahselt and Ownbey 1968). For example, hybrids of goat willow (*S. caprea*) and creeping willow (*S. repens*) had PG concentrations that were intermediate to those of their parental species (Hallgren et al. 2003). In contrast, hybrids of heart‐leaved willow (*S. eriocephala*) and silky willow (*S. sericea*) contained PG concentrations lower than that of the parental average concentration (Orians et al. 2000). Defenses in Booth's willow are uncharacterized, so we cannot determine which, if any, of these hybridization effects could be occurring across the Utah population.

Geographic population‐level differences in defense levels could also have been driven by differential selection pressures by browsers. This has been shown in multiple species (Castillo et al. 2014; Kato et al. 2017), including quaking aspen, where hares consumed less material from individuals from Alaskan populations of birch and aspen due to their higher chemical concentrations compared to sources from NE America (Bryant et al. 1994). A similar association may exist among the three Arizona willow populations, since historically, two different elk subspecies inhabited distinct regions between the willow populations (Harn 1958; Kohl et al. 2023). The vast geographic distances separating these populations may have facilitated an “island effect,” leading to evolutionary divergence between them. There were two factors that could have contributed to a reduction in defense traits within island plant genotypes compared to their mainland continental United States counterparts. One factor was the lack of mammalian herbivores from the islands, allowing for this genotypic divergence, and the other is related to differences in climatic conditions (Freedman et al. 2024).

## 5 Conclusions

Our findings offer valuable insights into the correlation between plant growth and the defense of Arizona willow. Analysis of both long‐term and short‐term protective measures suggests that these plants may not exhibit immediate plastic response to external changes, potentially necessitating an extended recovery period from persistent browsing pressure. Notably, our observations highlight the effectiveness of protective measures in fostering plant growth and lowering PG concentrations, potentially enhancing sexual reproduction, which could increase population sustainability. Further investigation is warranted to disentangle the intricacies of the trade‐off on plant growth and defense production, including exploring the effects of age, ontogeny, and dioecy on defense chemistry concentrations and susceptibility to browsing. Examining the time frame on plasticity response once browsing is removed is essential, as is delving deeper into the ODT dynamic within a common garden study. Understanding the dynamics of browsing on fitness in a sensitive species can help land managers make decisions that will best suit species success and survival.

## Author Contributions

**Shannon J. Lencioni:** data curation (lead), formal analysis (lead), funding acquisition (supporting), investigation (lead), methodology (equal), writing – original draft (lead), writing – review and editing (equal). **Ken Keefover‐Ring:** investigation (supporting), methodology (supporting). **Rob Massatti:** conceptualization (equal), funding acquisition (lead), project administration (equal), resources (lead), supervision (equal), writing – review and editing (supporting). **Liza M. Holeski:** conceptualization (equal), funding acquisition (supporting), investigation (supporting), methodology (equal), project administration (equal), resources (equal), supervision (equal), writing – review and editing (lead).

## Ethics Statement

The authors have nothing to report.

## Conflicts of Interest

The authors declare no conflicts of interest.

## Supporting information


Data S1.

## Data Availability

Data has been made available on Dryad Digital Repository doi:10.5061/dryad.1g1jwsv66.

## Appendix A

**TABLE A1:** Site climate characteristics within populations.

Population	Site name	Number samples	Number subsites	Mean height (cm)	Mean elev. (cm)	MAP (mm)	MAT (°C)	MAT (°C)	*T*max (°C)
Arizona	White Mtns	50	9	127.39	2826	830.84	5.2	−2.6	12.9
New Mexico	Cumbress	36	4	103.83	3118	826.39	2.5	−4.4	9.4
	Cabresto	54	6	35.48	3186	652.08	3.4	−3.2	10
	San Pedro	9	2	67.78	3044	887.61	4.5	−2.1	11.1
	Pecos	30	4	41.97	3407	893.24	2.9	−3.2	9.1
Utah	Dixie	36	4	73.50	3129	797.01	2.7	−3.5	8.8
	Fishlake	30	5	87.78	2982	859.22	3.6	−2.3	9.5

*Note:* Mean elevation was calculated by averaging the elevation taken at each collection site. Climate data were extracted from Prism Climate Group using recent 30‐year averages for the geographic middle of each collection site.

**TABLE A2:** Model Selection for looking into interaction effects by total and individual PGs, AIC score provided for each model.

Model	Total PGs	Salicin	Salicortin	HCH‐salicortin	Unk 1	Unk 2
log(Height) = Browse + PG + Pop + Cage + (1|site/subsite)	369.14	371.59	369.69	365.03	366.97	365.01
log(Height) = Browse + PG + Pop + Cage + Browse*PG + (1|site/subsite)	388.32	395.61	390.98	**369.98**	364.21	368.52
log(Height) = Browse + PG + Pop + Cage + Browse*Pop + (1|site/subsite)	366.91	369.35	367.39	361.53	363.72	363
log(Height) = Browse + PG + Pop + Cage + Browse*Cage + (1|site/subsite)	374.8	377.5	375.5	370.37	371.82	370.76
log(Height) = Browse + PG + Pop + Cage + PG*Pop + (1|site/subsite)	392.08	385.35	390.92	372.99	**374.19**	**365.1**
log(Height) = Browse + PG + Pop + Cage + Pop*Cage + (1|site/subsite)	370.61	372.77	371.09	366.45	368.44	366.22
log(Height) = Browse + PG + Pop + Cage + PG*Cage + (1|site/subsite)	380.86	377.51	380.76	369.55	375.44	365.01

*Note:* Only one interaction model had one significant interaction, though had a significantly higher AIC score than simpler model. (Moderate Browse Severity: HCH‐salicortin had a significant effect where estimate = −0.269, *p* = 0.0287; Unknown 1 had a significant interaction effect within a 90% CI with PG and Population, where Unk1: New Mexico estimate = 0.194, *p* = 0.0625; Unknown 2 had a significant interaction effect within a 90% CI with PG and Population, where Unk2: Utah estimate = −0.519, *p* = 0.0668). Significant interactions in bold.
